# Exploring the alpha desynchronization hypothesis in resting state networks with intracranial electroencephalography and wiring cost estimates

**DOI:** 10.1038/s41598-017-15659-0

**Published:** 2017-11-15

**Authors:** Jaime Gómez-Ramírez, Shelagh Freedman, Diego Mateos, José Luis Pérez Velázquez, Taufik A. Valiante

**Affiliations:** 1 The Hospital for Sick Children, Neurosciences and Mental Health program, Toronto, Canada; 2 Concordia University, Montreal, Canada; 3 Toronto Western Hospital, Krembil Research Institute, Toronto, Canada

## Abstract

This paper addresses a fundamental question, are eyes closed and eyes open resting states equivalent baseline conditions, or do they have consistently different electrophysiological signatures? We compare the functional connectivity patterns in an eyes closed resting state with an eyes open resting state to investigate the alpha desynchronization hypothesis. The change in functional connectivity from eyes closed to eyes open, is here, for the first time, studied with intracranial recordings. We perform network connectivity analysis in iEEG and we find that phase-based connectivity is sensitive to the transition from eyes closed to eyes open only in interhemispheral and frontal electrodes. Power based connectivity, on the other hand, consistently discriminates between the two conditions in temporal and interhemispheral electrodes. Additionally, we provide a calculation for the wiring cost, defined in terms of the connectivity between electrodes weighted by distance. We find that the wiring cost variation from eyes closed to eyes open is sensitive to the eyes closed and eyes open conditions. We extend the standard network-based approach using the filtration method from algebraic topology which does not rely on the threshold selection problem. Both the wiring cost measure defined here and this novel methodology provide a new avenue for understanding the electrophysiology of resting state.

## Introduction

The view of the brain as a reflexive organ whose neural activity is completely determined by incoming stimuli is challenged by the “intrinsic” or spontaneous view of the brain. Nevertheless, the exact implications of resting state for brain function are far from clear^1,2^. Mandag and colleagues^3^ argue for the reconceptualization of resting state as an independent variable (brain’s input) to a multidimensional activity modulator. The emerging field of functional connectomics relies on the analysis of spontaneous brain signal covariation to infer the spatial fingerprint of the brain’s large-scale functional networks. While there is growing interest in the brain’s resting state, supported by evidence for persistent activity patterns in the absence of stimulus-induced activity (e.g. default mode network)^4^, there lacks a definite recommendation about whether resting state data should be collected with participants’ eyes open or closed. If stimulus-induced activity is indeed, at least in part, predetermined by the brain’s intrinsic activity (i.e. resting state activity), it follows that we cannot understand one without the other. The more we know about the electrophysiological underpinnings of resting state, both with eyes closed and eyes open, the better equipped we will be to understand brain dynamics, including both intrinsic activity and the processing of stimuli.

The orthodox approach to understanding brain function relies on the view of the brain as an organ that produces responses triggered by incoming stimuli, which are delivered at will by an external observer. This idea has been challenged by the complementary view of the brain as an active organ, with intrinsic or spontaneous activity^5–7^. Crucially, the brain’s intrinsic activity both shapes and is shaped by external stimuli. While there has been some controversy concerning the ecological relevance of studying a default or resting condition^8,9^, the empirical evidence for intrinsic brain activity is conclusive^10,11^.

Despite the ever increasing importance of resting-state functional connectivity (a quick search on PubMed shows 2,742 papers with the term “resting state” in the title at the time of the writing), it remains underutilized in clinical decision making^12^. A rationale for this needs to be found with both conceptual and methodological basis. First and foremost, the term resting-state is a misnomer, as a matter of fact, the brain is always active, even in the absence of an explicit task, or external stimuli. Cognitive task-related changes in brain metabolism, measured with PET, account for a mere 5% or less of the brain’s metabolic demand^13^. Second, the resting state literature, from its inception, is eminently based on the analysis of low frequency fluctuations of the BOLD signal measured using fMRI, alone or in combination with EEG and PET^14,15^. Third, these techniques suffer from suboptimal temporal and/or spatial resolution and the haemodynamic or metabolic activity measured in fMRI and PET are proxy measures for the electrophysiological activity. Fourth, there is a lack of consensus in the literature regarding whether resting state data should be collected while the participant has their eyes open, closed, or fixated. See^16^ for non-significant between-condition differences in resting state networks and^17^ for an antagonistic view. This paper attempts to better understand the brain’s resting state by characterizing the two most common baseline conditions in neuropsychology, eyes closed and eyes open, using intracranial electroencephalogram recordings. Note that intracranial electroencephalography, iEEG, and electrocorticography, ECoG, are here used indistinctly.

Previous studies have identified a reduction in the number of connections when the eyes closed condition is compared to the eyes open condition, in the alpha band^18,19^. This is known as “alpha desynchronization”. Using EEG, Barry and colleagues^19^ found that there are electrophysiological differences -topography as well as power levels- between the eyes closed and eyes open resting states. A higher degree of alertness caused by opening one’s eyes is associated with the attenuation of alpha rhythm, which is supplanted by desynchronized low voltage activity^20^. Geller and colleagues^21^ found that eye closure causes a widespread low-frequency power increase and focal gamma attenuation in the human electrocorticogram. However, although these studies explicitly conclude that eyes open and eyes closed are different baseline conditions, they do not provide a method for comparing the functional connectivity patterns elicited by either of the two conditions against a common criterion.

Shedding some light on the problem, this paper examines whether the eyes closed and eyes open resting states are equivalent baseline conditions by analyzing the differences between the two, using a filtration approach that extends the standard network-based approach of using a fixed threshold to obtain the adjancency matrix from the correlation matrix. In a filtration method, a set of networks are built for a large number of thresholds, overcoming the threshold selection problem of building a graph from a correlation matrix. This allows us to explore, systematically and bias free, the electrophysiological underpinnings of resting state with intracranial electroencephalogram data.

First, we perform power and phase based connectivity analysis to asses whether the connectivity patterns calculated from intracranial recordings are able to differentiate between the two conditions. Additionally, we exploit the excellent temporal and spatial precision of ECoG to calculate the wiring cost for the connectivity maps.

Second, we investigate whether network topological properties have enough statistical power to be used as a feature/covariate to distinguish between the eyes closed and eyes open conditions. Finally, we extend the network theory based results, borrowing from algebraic topology, to perform a filtration method to study the dynamics of the network topologies for a large number of thresholds.

## Materials and Methods

### Participants

**Figure 1:**
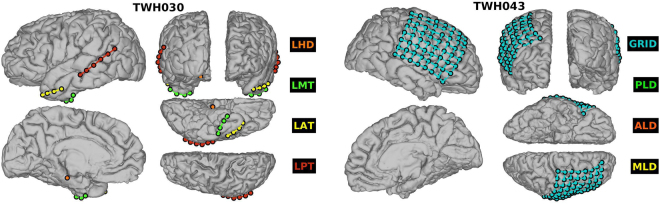
Schematic of the electrode implant for two participants. (**left**) Participant 5 with bitemporal implant, 36 electrodes total, 18 in each hemisphere, including a strip of 6 electrodes in the posterior temporal, strip of 4 electrodes in the medial temporal and the anterior temporal and 4 depth electrode contacts in the hippocampus. (**right**) Participant 13 with Grid and depth electrodes. In blue the Grid of 64 contacts (8 × 8 matrix), the depth electrodes are not visible in this figure.

The intracranial electroencephalography recordings were collected at the Toronto Western Hospital (Toronto ON, Canada). Our research protocol was approved by the University Health Network Research Ethics Board and informed consent was obtained from the participants. All methods were performed in accordance with the relevant guidelines and regulations. Informed consent was obtained from all patients. Eleven participants (6 female) with pharmacologically-refractory mesial temporal lobe epilepsy underwent a surgical procedure, in which electrodes were implanted subdurally on the temporal lobe and stereotaxic depth electrodes were implanted in the hippocampi or other deep structures (Fig. 1). For each patient, electrode placement was determined to best pinpoint the origin of seizure activity. In addition to electrodes implanted in the temporal lobe, including depth electrodes in the hippocampi, some patients had electrodes implanted in frontal, interhemisphic and the cortical convexity (see Table 1). The electrode implants are thus not identical for all participants, though they tend to overlap in the mesial temporal lobe epilepsy (MTLE) sensitive regions. This limits the ability to directly compare the wiring cost or other network properties among participants. However, we can still compare and generalize from participants by examining the difference between the two conditions. For example, in order to compare the functional connectivity pattern of two participants, one with a grid in the left cortex and another with depth electrodes in the hippocampus and temporal areas, we calculate the difference between network parameters from eyes closed to eyes open, within each participant.

**Table 1:** ID, sex, age, laterality and type of implant.

Patient	Sex	Age	Laterality	Channels
5	F	45	B	H, T
6	M	32	B	H, T, F, IH
7	M	41	B	H, T, F, IH
10	F	24	B	FP, IH, F
11	F	29	B	H, T
12	M	49	B	H, T
13	F	18	B	Grid, D
15	F	29	R	H, FP, F, IH, T
16	F	37	B	H, T
17	M	28	L	Grid, D
18	M	25	R	H, FP, F, T

The laterality can be bilateral (B), left (L) and right (R). The location of the electrodes fall under the following categories: hippocampus (H), temporal (T), frontal (F), interhemispheral (IH), frontal polar (FP), Grid, and depth (D; different from hippocampus).

### Resting state conditions

To assess resting-state activity for both the eyes closed and and eyes open conditions, participants were asked to relax and rest quietly in their hospital bed, in a semi-inclined position. First, they were asked to close their eyes for three minutes and then asked to keep their eyes open for another three minutes. Each session was recorded with real-time monitoring of the intracranial electroencephalography and continuous audio and video surveillance.

ECoG recordings allow us to simultaneously study both fast and slow temporal dynamics of the brain at rest, that is, not engaged in tasks prescribed by the experimenter. Freeman and Zhai^22^ have shown that the resting ECoG has low-dimensional noise, making resting state an optimal starting point for defining and measuring both artifactual and physiological structures emergent in the activated electrophysiological signals. Importantly, ECoG signals covary in patterns that resembled the resting state networks (RSN) found with fMRI^23^.

### iEEG acquisition

Continuous iEEG data were recorded in an unshielded hospital room using NATUS Xltech digital video-EEG system. Commercially available depth electrodes and subdural electrodes were used to collect continuous iEEG recordings. Common reference and ground electrodes were placed subgalealy at a location distant from any recording electrodes with contacts oriented toward the dura. Electrode localization was accomplished by localizing the implanted electrodes on the postoperative computed tomography (CT) scan using the Matlab toolbox iELVis for localizing and displaying human intracranial electrode data^24^. Subdural electrodes were arranged in strip or grid configurations, with an inter-electrode spacing of 10 mm. The location of the electrode implants was not identical across patients, however, all participants had depth electrodes, mostly in the hippocampi (Table 1).

### Signal processing

Signals were filtered online using a high-pass (0.1 cutoff frequency) and an anti-aliasing low-pass filter. Offline filtering using Matlab in house-scripts, consisted of a high-pass and low-pass filter at 0.5–70 Hz and a notch filter applied at 60 Hz to remove electrical line noise.

To extract power and phase estimates of time-varying frequency-specific band, the ECoG signals were convolved with complex-valued Morlet wavelets. The wavelet convolution transformed the voltage trace at each electrode to obtain both instantaneous power and phase trace for each frequency. The wavelet length was defined in the range of −1 to 1 seconds and was centered at *time* = 0 (in doing so we guarantee that the wavelet has an odd number of points). We used a constant number of wavelet cycles (7). This number was chosen since we have long trial periods (3 minutes) in which we expect frequency-band-specific activity and a large number of cycles (from 7 to 10) to facilitate identifying temporally sustained activity^25^.

Of note, it is also possible to use a number of wavelet cycles that changes as a function of frequency, to adjust the balance between temporal and frequency precision as a function of the frequency of the wavelet. Thus, there is a trade-off between temporal and frequency precision. Since we are processing long epochs, we favour frequency over time precision and therefore chose to use a large number of wave cycles.

### Connectivity measures

We are interested in calculating the wiring cost associated with the functional connectivity map defined upon the electrodes’ spatial location. Measures of correlated activity are not real measures of “connectivity”. Here we use the term connectivity because it is the standard nomenclature, but a caveat on the dangers of assuming equality between correlated activity and connectivity is worth mentioning. Functional connectivity is calculated using both power-based and phase-based measures. For power-based we calculate Spearman’s correlation, while for phase-based connectivity we calculate two different measures - phase-lag index (PLI)^26^ and intersite phase clustering (ISPC). Note that ISPC represents the clustering in polar space of phase angle differences between electrodes resulting from the convolution between a complex wavelet and the signal and is also referred in the literature as R^25^.

Next, we briefly outline the three connectivity measures used. First, we describe power-based connectivity and next phase-based connectivity for the phase lag index and intersite phase clustering measures.

#### Power-based connectivity

To calculate the correlation coefficients for power time series from any two electrodes in the same frequency, we perform time-frequency decomposition using wavelets to then compute the Spearman correlation coefficient between the power time series of the two electrodes. To increase the signal to noise ratio, we segment the data into non-overlapping windows of 5 seconds, compute Spearman’s correlation coefficient for each segment, and then average the correlation coefficients together.

The Spearman’s correlation is the Pearson correlation of the data previously rank-transformed. Formally, the Spearman correlation of two channels *x* and *y* whose power time series values have been rank-transformed is:

1 $${r}_{xy}=\frac{{\sum }_{t=1}^{n}(x(t)-\bar{x})(y(t)-\bar{y})}{\sqrt{{\sum }_{t=1}^{n}{(x(t)-\bar{x})}^{2}{\sum }_{t=1}^{n}{(y(t)-\bar{y})}^{2}}}$$

It is of note that power-based correlation coefficients range from −1 to 1. To have a more normal looking distribution, it is preferable to perform a Fisher-Z transformation. It ought to be noted that power correlation is not limited to the kind of instantaneous correlations performed here, for example, cross correlation detects peak connectivity between two time series as a function of time lag.

#### Phase-based connectivity

We calculate phase-based connectivity using two different measures, intersite phase clustering (ISPC) and the phase-lag index (PLI). The ISPC measures the clustering in polar space of phase angle differences between electrodes and is given by the equation:

2 $${ISP}{{C}}_{f}=|{n}^{-1}\sum _{t=1}^{n}{\exp }^{i({\varphi }_{x(t)}-{\varphi }_{y(t)})}|$$

where n is the number of time points and *ϕ*_*x*_ and *ϕ*_*y*_ are the phase angles from electrodes *x* and *y* at a given frequency *f*. Note that this measure is sensitive to volume conduction. For example, when the phase differences are not uniformly distributed, but clustered around 0 or *π* in polar space, much of the apparent connectivity between these electrodes might be due to volume conduction.

There are several phase-based connectivity measures that ignore the 0 − *π* phase-lag connectivity problem, e.g., imaginary coherence^27^, phase-slope index^28^, phase-lag index^26^ and weighted phase-lag index^29^. Although these measures are designed to be insensitive to the linear mixing of uncorrelated sources, in some cases they may still be susceptible to source mixing^30^.

Phase lag index measures the extent to which the distribution of phase angle differences is more to the positive or to the negative side of the imaginary axis on the complex plane. That is, it tells us whether the vector of phase angle differences are pointing up or down in polar space. The idea is that if spurious connectivity is due to volume conduction, the phase angle differences will be distributed around zero radians. It follows that non-volume conducted connectivity will produce a distribution of phase angles that is predominantly on either the positive or the negative side of the imaginary axis. Note that here, contrary to ISPC, the vectors are not averaged, instead it is the sign of the imaginary part of the cross spectral density that is averaged:

3 $${PL}{{I}}_{xy}=|{n}^{-1}\sum _{t=1}^{n}{sgn}({imag}({S}_{xy}(t)))|$$

where *imag* is the imaginary part of *S*_*xy*_(*t*), or cross-spectral density between channels *x* and *y* at time *t*. The *sgn* function returns +1, −1, or 0.

Phase coherence measures are highly influenced by volume conduction^31^. PLI, on the other hand, was designed to tackle this problem. As shown by Stamm and colleagues, PLI is not particularly sensitive to zero-lag correlations and is less sensitive to volume conducted signals and common reference issues^26^. Later on, Peraza and colleagues^30^ have shown that PLI is not entirely invariant to volume conduction. In a simulation study, they found that PLI-based connectivity networks show more small worldness (higher cluster coefficient) than random networks. However, for non-volume conduction, PLI-based networks are close to random networks, indicating that the high clustering shown for PLI is caused by volume conduction.

To recapitulate, ISPC captures the clustering of the phase angle difference distribution and PLI the phase angle directions. ISPC can be influenced by changes in power and is maximally sensitive to detecting connectivity, regardless of the phase angle differences. Intracranial EEG is less sensitive to volume conduction problems than other electrophysiological techniques (EEG and MEG). Thus, by calculating phase-based connectivity with both ISPC and PLI, we expect to clarify the properties of both measures for the analysis of the iEEG signal.

#### Wiring cost

Now that we have described how functional connectivity is obtained, we continue by describing how to calculate the wiring cost between any pair of electrodes. The idea behind this measure is to exploit the location of the signal to provide a measure of the wiring cost of having two electrodes coupled, that is, statistically correlated, by any of the connectivity measures highlighted above. The wiring cost is nothing more than the connectivity matrix weighted by the euclidean distance between the electrodes. To calculate the wiring cost, we need then two matrices, the distance matrix ${D}_{ij}=\Vert ({x}_{i},{y}_{i},{z}_{i}),({x}_{j},{y}_{j},{z}_{j})\Vert$ which captures the Euclidean distance between any two electrodes physically located in Cartesian coordinates (*x*_*i*_, *y*_*i*_, *z*_*i*_) and (*x*_*j*_, *y*_*j*_, *z*_*j*_) and the functional connectivity matrix. Thus, the computation of the wiring cost *W* combines the physical distance matrix *D* and a functional connectivity matrix *F*. While there is one phyisical distance matrix *D* for each participant, we calculate the functional connectivity matrix *F* using three different criteria - ISPC, PLI and the Spearman correlation of power time series.

The pairwise wiring cost for a distance matrix of electrodes *D* and functional connectivity matrix *F* calculated at frequency *f* is calculated as:

4 $$W(f)=D\,\,.\ast \,\,F(f)$$

**Figure 2:**
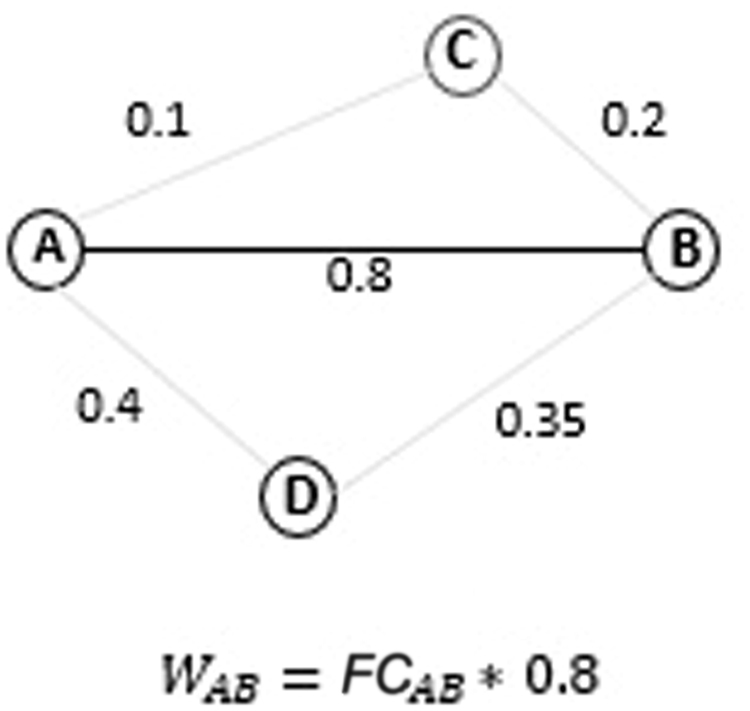
The nodes represent electrodes and the edges the correlation between the nodes. The wiring cost between electrodes *A* and *B* is calculated as the product between the Euclidean distance between the nodes and the functional connectivity. Thus the wiring cost between two nodes A, B is the functional connectivity value weighted by the distance, *W*_*AB*_ = *D*_*AB*_ * *FC*_*AB*_.

Thus, the pairwise wiring cost of two electrodes is directly proportional to the distance and the correlation. The further away and the stronger the correlation, the larger the wiring cost (Fig. 2).

### Network analysis

The correlation matrices can be converted into adjacency matrices and then into undirected graphs with the direct application of a threshold. The choice of the threshold specifies the relationship between two electrodes, two electrodes are connected when the correlation is within a certain threshold. Thus, two electrodes are connected when the correlation is larger than the threshold.

**Figure 3:**
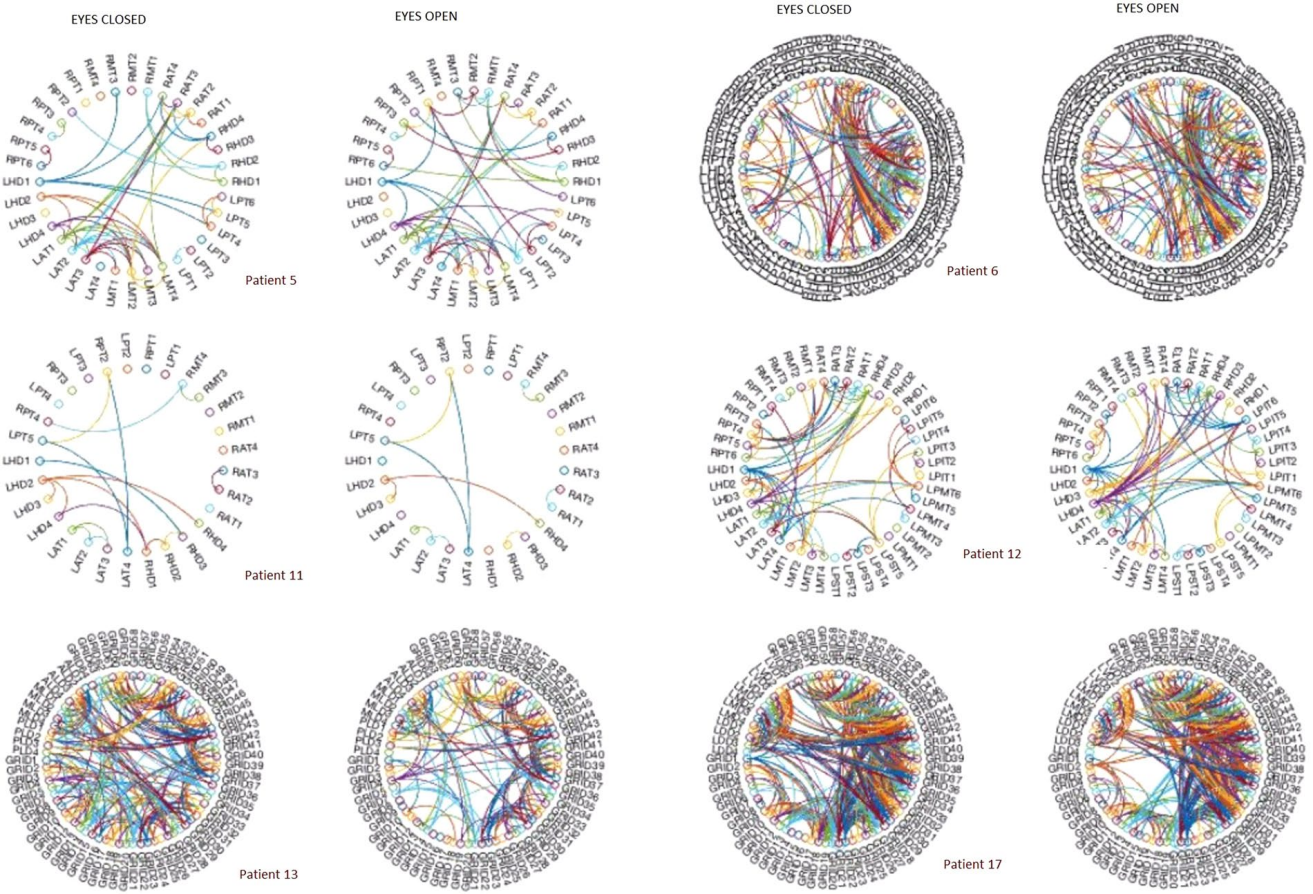
The figure shows the power-based connectivity network for 6 subjects for eyes closed and eyes open in the alpha band. The threshold used is *t* = *μ* + *σ*. Top left and clockwise, subjects 5, 6, 11, 12, 13 and 17. The first and third columns depict the network in eyes closed, and columns 2 and 4 in eyes open. Network property changes can be directly observed, for example, for patient 11 (second row, columns 1 and 2) the number of edges decreases from eyes closed to eyes open, mainly due to a loss of connections between the right and the left hippocampi.

Figure 3 shows the binary or unweighted networks that result from thresholding the power based correlation matrices in the alpha band. The threshold of choice is equal to the mean plus one standard deviation. We build the network connectivity for each subject and condition in the frequency band to then calculate an extensive set of network metrics including clustering, transitivity, path length, and number of components.

It is important to note that although the majority of subjects have electrodes in temporal areas and the hippocampi, the location of the electrodes varies substantially from one subject to another and the subjects’ networks are not directly comparable. For example, a subject with a grid of 64 contacts with a separation of 1 cm will necessarily have a larger clustering coefficient than a subject with bitemporal electrodes, and by the same token, the average path length in stereotactically implanted electrodes will be larger than in the grid.

In order to avoid this limitation, we study the difference in the network metrics between conditions for each subject. In this way, we can compare the variations in network topology for the two conditions across subjects.

### Persistent homology

A not less important limitation is that we obtain very different networks depending on the significance level (threshold) we use. This is problematic, particularly when the underlying system it not scale invariant. Small world and clusterness are joint measures and can change drastically depending on the choice of threshold^32^. Furthermore, by adopting a threshold, we may be loosing important information, for example, it may occur that some small-scale features are noise artifacts while other are critically important^33,34^.

Algebraic topology^35^ provides a language and a methodology to overcome these limitations. It presents a multiscale framework able to deal with the threshold selection problem. In the standard approach, in order to study the topological properties of functional connectivity networks we need to consider a threshold, which once applied to the connectivity matrix, will produce a binary graph from which network properties such as clustering, small world, characteristic path length and others can be measured. The selection of the threshold is, however, arbitrary, and the resulting network depends entirely upon that choice.

We overcome this limitation by following a filtration method used in algebraic and computational topology^36,37^, in which rather than having one threshold, we build a vector of thresholds, containing all possible threshold values between the two extremes (minimum and maximum connectivity values). For example, for the matrix *F*, of dimension *n* × *n* we obtain the threshold vector *T* with *n*^2^ elements bounded between the minimum and maximum of *F*, *T* = [*min*(*W*), *max*(*W*)].

A set of binary networks is then obtained by thresholding the wiring cost matrix for each possible threshold. Specifically, the binary matrix *B*_*τ*_ for the threshold *τ* and functional connectivity matrix *F* is such that *B*_*τ*_(*ij*) = 0 if the correlation between electrodes *i*,*j* is less than the threshold, *B*_*τ*_(*ij*) < *τ*, otherwise *B*_*τ*_(*ij*) = 1. Thus, for each threshold value *τ* ∈ *T*, we obtain a binary network and the resulting set of networks is comprised at the two extremes of the spectrum by the disconnected graph *B*_*τ*_(*V*, ∅), produced when applying the threshold *τ* = *min*(*W*) and the full graph *B*_*τ*_(*V*, *E*(*W*)) resulting from applying the threshold *τ* = *max*(*W*). Importantly, the set of binary networks has an internal structure that progressively increases until it becomes a fully connected network.

## Results

First, we study the statistical significance for the two conditions, eyes closed and eyes open, using the correlation matrix for power and phase based connectivity in the alpha band.

The effect of a higher degree of alertness (going from eyes closed to eyes open) for the various regions of interest for power-based connectivity in the alpha band is shown in Table 2. All the patients (11/11) have at least one electrode with a power-based connectivity pattern that is statistically significant for the two conditions.

**Table 2:** Statistical significance for power-based connectivity in the alpha band calculated for a 95% confidence interval.

Patient	H	T	F	IH	Grid	D
5	—	p = 0.0131(*)				
6	—	p = 0.0128(*)	p = 0.013(*)	p = 0.0312(*)		
7	p = 0.027(*)	p = 5.3506(**)	—	p = 0.0128(*)		
10			p = 0.0166(*)	p = 0.0113(*)		
11		p = 0.0062(**)			p = 0.0248(*)	
12	p = 0.0175(*)	p = 0.0058(**)				
13					p = 0.018(*)	—
15		p = 0.0017(**)	—	p = 0.0011(**)		—
16		p = 0.0244(*)				—
17					p = 0.0059(**)	
18		p = 0.0066(**)	p = 0.0056(**)	p = 0.0137(*)		—

The p-value is displayed when *p* < 0.05 (* when *p* < 0.05 and ** when *p* < 0.001), — denotes a non rejection of the null hypothesis or *p* > 0.05. For example, subject 5 has electrodes in the hippocampi (H) and in the temporal lobe (T) of which only electrodes in the temporal lobe reject the null hypothesis - that the mean power-based correlation in eyes closed and eyes open are not significantly different. All patients had at least one channel that was statistically significant, with a total of 84 channels with *p* < 0.05 in the alpha band.

Tables 3 and 4 show the statistical significance analysis for phase-based (ISPC and PLI) connectivity in the alpha band. Phase based connectivity demonstrated a statistically significant difference between the two conditions in interhemispheric and frontal electrodes for only 2 subjects (2/11). Depth, hippocampal and temporal electrodes do not show statistically significant differences between conditions. This is in agreement with EEG studies that show a decrease in alpha activity across the entire cortex in response to visual stimulation^19^.

**Table 3:** Statistical significance of phase ISPC-based connectivity in the alpha band.

Patient	H	T	F	IH	Grid	D
7	—	—	p = 0.0274(*)	p = 0.0294(*)		
10			p = 0.0224(*)	p = 0.0149(*)		

Only 2 subjects out of 11 have one channel or more with statistical significance.

**Table 4:** Statistical significance of phase PLI-based connectivity in the alpha band.

Patient	H	T	F	IH	Grid	D
7	—	—	p = 0.0274(*)	p = 0.0247(*)	—	—
10			p = 0.0406(*)	p = 0.0087(**)		
11	—	p = 0.0219(*)				

Only 3 subjects out of 11 have one channel or more with statistical significance.

### Power based connectivity (network topology)

Based on the previous results, we focus on power-based connectivity to study the network topology difference in the two conditions, eyes closed and eyes open. In order to study the topological properties we need to build the network from the correlation matrix. The procedure is quite straight forward, from the correlation matrix, we apply a threshold, in this case, the mean plus one standard deviation, *t* = *μ* + *σ*, to obtain the adjacency matrix, which can be equally represented as a graph. Figure 3 shows the connectivity network for 6/11 subjects for threshold *t* for both eyes closed and eyes open.

**Figure 4:**
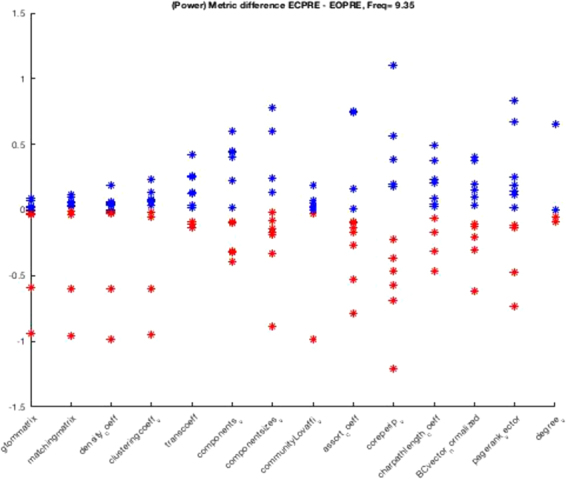
The figure represents the difference between a number of network metrics for power-based connectivity in the alpha band in eyes closed minus eyes open. In the x-axis are plotted the network metrics, namely and from left to right: Generalized topological overlapping measure, Matching index, Density coefficient, Clustering coefficient, Transitivity coefficient, Number of connected components, Size of connected components, Community structure, Assortativity coefficient, Core/periphery structure, Characteristic path length, Eigenvector centrality normalized, Page rank centrality, and Degree. In the y-axis are plotted the difference in value between the two conditions for each metric, when the difference is positive the dot is plotted in blue, otherwise in red. The number of points plotted for each metric is equal to the number of subjects. In none of the network metrics do we observe a strict increase (all red points) or strict decrease (all blue dots) of the topological properties studied. However, since the electrodes implants are not spatially coincident, it is impossible to draw any overarching conclusion about how the network is affected from eyes closed to eyes open.

For a quantitaive analysis on the topological changes in the two conditions we calculate the network metric differences calculated for power-based connectivity in the alpha band as shown in Figure 4. The x-axis represents different network metrics and the y-axis represents the difference between the network metric, for example clustering (fourth point in the x-axis), in going from eyes closed to eyes open. When the difference is positive, for example, clustering in eyes closed is larger than in eyes open, the dot is blue, otherwise red. While this approach has the potential to help us understand how the topological properties of the connectivity network are affected between the two conditions, there is an important caveat to keep in mind. Although all the subjects in our data set tend to have electrodes in temporal areas and the hippocampi, the location of the electrodes varies substantially from one subject to another and the subjects’ networks are not directly comparable. It is, however, possible to overcome this limitation if we study on a single-subject basis the network properties for a set of thresholds. Thus, rather than assuming that the threshold is fixed, we build a large number of networks, as many networks as thresholds. In this way, we create a population of networks for each subject and condition from which it is possible to derive statistics. This is described in Section 2.9.

### Power based connectivity (filtration method)

To acquire a qualitative understanding of both conditions, eyes closed and eyes open, in terms of the wiring cost, we need to perform statistics with the distribution of binary networks obtained from using a large number of thresholds. The null hypothesis is that the effect of eyes closed is indistinguishable from the effect of eyes open for wiring cost. We extend the previous approach that consists of building the resulting network from applying the threshold of choice to the connectivity matrix, to building a set of networks with one for each possible threshold from the same connectivity matrix. Crucially, by removing the initial assumption of a fixed threshold which is necessarily ad hoc, we can study the network dynamics of the n resulting networks, one for each threshold in the n-dimensional vector of thresholds.

**Figure 5:**
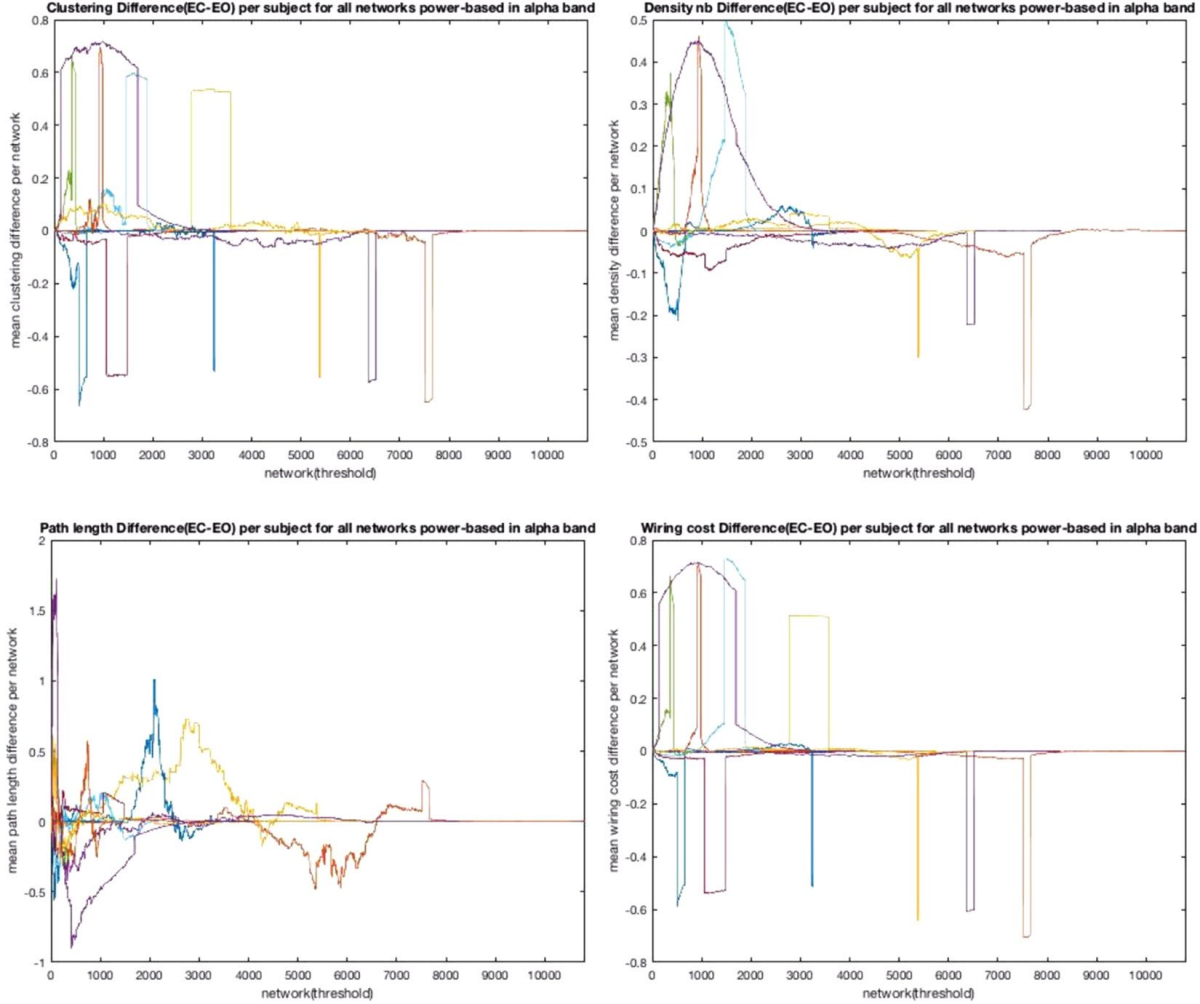
The figure (top left and clockwise) shows the difference in clustering coefficient, density, characteristic path length and wiring cost between eyes closed and eyes open in the alpha band. The x-axis represents a network built upon applying a threshold. The difference always converges at zero, for some large threshold in which the network is fully connected (clique). In that case, the networks are identical for the two conditions and the difference is therefore 0.

Figure 5 shows the difference in clustering coefficient, density of edges, characteristic path length and wiring cost between eyes closed and eyes open.

We perform a test of statistical significance for the four network properties highlighted in Figure 5. The results are shown in Table 5. In 4/11 subjects all the network metrics show a statistically relevant difference between the two conditions. The network metric with the best score in differentiating between eyes closed and eyes open is the wiring cost, in 8/11 subjects.

**Table 5:** The table shows the statistical significance test for four network metrics, clustering coefficient, density (ratio between actual connections and potential connections), path length and wiring cost.

Patient	Clustering	Density	Path length	Wiring Cost
5	**	**	**	**
6	p = 0.107	p = 0.192	p = 0.271	p = 0.336
7	p = 0.768	p = 0.546	p = 0.101	p = 0.601
10	**	**	**	**
11	**	**	p = 0.311	**
12	**	**	p = 0.878	**
13	**	**	**	**
15	p = 0.609	p = 0.278	**	p = 0.991
16	p = 0.599	**	**	**
17	**	p = 0.0328	p = 0.957	**
18	**	**	**	**

Clustering coefficient differentiates between eyes closed and eyes open in 7/11 subjects, Density in 7/11 subjects, characteristic path length in 6/11 and wiring cost in 8/11 patients.

## Discussion

The brain is energy hungry, it amounts to only the 2% of the weight of the body, but takes up to 20% of the body’s metabolic demand. Yet, as with all physical systems, the brain has energy limitations. Ramón y Cajal was the first to postulate the laws of conservation for time, space and material^38^. It follows that there is a strong pressure for efficient use of resources, for example the minimization of the wiring cost at axonal, dendritic and synaptic levels. Longer connections, and those with greater cross-sectional area, are more costly because they occupy more physical space, require greater material resources, and consume more energy per connection. Networks that strictly conserve material and space (e.g. lattice) will likely pay a price in terms of conservation of time: it will take longer to communicate an electrophysiological signal between nodes separated by the longer path lengths that are characteristic of lattices^39^. There are trade-offs between biological cost and topological value.

Functional connectivity analysis from EEG data provides an explanation for alpha desynchronization in terms of the number of connections i.e., the number of connections decreases when one’s eyes are open compared to closed. It is worth noting that the term desynchronization is defined in the literature quite vaguely, and used to mean very different things. Synchronization sometimes refers an to increase in band power in some frequency band (e.g. alpha) and conversely, desynchronization is also associated with a loss of power in the frequency band of interest. Stam *et al*.^40^ provide an alternative approach to desynchronization of the alpha rhythm, which is characterized as an increase in the irregularity of the EEG signal. The EEG irregularity is quantified with the acceleration spectrum entropy (ASE), which is the normalized information entropy of the amplitude spectrum of the second derivative of a time series.

This study investigates the electrophysiological signatures that characterize eyes closed and eyes open resting states in patients diagnosed with mesial lobe epilepsy, taking advantage of the unmatched spatio-temporal properties of iEEG. Power and phase based connectivity analysis were performed for both conditions in the alpha band, to investigate the alpha desynchronization hypothesis. Alpha desynchronization, or the alpha blocking response to eye opening was originally reported by Berger in 1929. Alpha suppression is produced by an influx of light, other afferent stimuli and mental activity^41^. Alpha rhythm is the EEG correlate of relaxed wakefulness, best obtained while the eyes are closed^20^.

The wiring cost, as defined here, combines the physical distance between electrodes and the statistical correlation and takes full advantage of the spatial resolution of the ECoG signal. Specifically, the local wiring cost of two electrodes represents the product between the distance and the correlation value. The combination of functional connectivity and distance networks allows us to quantify the wiring cost for the two conditions under study -eyes closed and eyes open. The rationale behind this approach is that the wiring cost might explain, at least in energy minimization terms, why, among all possible configurations, some functional connectivity patterns are selected rather than others. We mathematically define the wiring cost for a given connectivity pattern in Equation 4.

We do not find compelling evidence for alpha desynchronization in phase-based connectivity analysis (except for interhemispheral and frontal electrodes). Power-based connectivity, on the other hand, is a more consistent predictor of alpha desynchronization, in particular within temporal electrodes. We find that the wiring cost does a better job in differentiating between eyes closed and eyes open than network metrics such as characteristic path length, clustering, or the edge density.

To investigate the loss of connectivity predicted by the alpha desynchronization hypothesis without relying on the adoption of a network threshold, we calculated the distribution of network property values associated with the connectivity matrix derived from a threshold vector bounded by the minimum and maximum functional connectivity values. We find that the location of the electrodes is the most important factor to be considered when studying the alpha desynchronizationin ECoG.

Although intracranial electroencephalography has unmatched spatial and temporal specificity, it may not be the optimal method for studying macroscopic aspects of the human brain. This study has the limitation that the electrode implants tend to be located in the seizure sensitive temporal lobe and leave untouched occipital and parietal lobes. A complementary model system for the study of the wiring cost difference between two connectivity patterns would be EEG or fMRI, in which the signal source is regularized in a common brain volume template. However, these techniques are limited by the source reconstruction problem, which is not as problematic in iEEG.

Ideally, this study would have used randomization of the two conditions -eyes closed and eyes open- altering the order. In the alpha blocking response to eye opening initially described by Berger (Berger’s effect), also called alpha desynchronization, there is a specific sequence – eyes closed precedes eyes open- and this is the sequence that we have used. It is however possible to go beyond the alpha blocking response to a more general study of the electrophysiological signatures of eyes open and eyes closed. This would require randomization and will be studied in future work in which we perform an intervention between eyes closed and eyes open, alternatively.

The results here obtained can be of interest to resting state (sleep, awake), task-based and pathological conditions, for example in epileptic seizures. In a forthcoming study, we show that the wiring cost increases dramatically in the ictal period compared to the pre-ictal period.

This work is a step forward in understanding the electrophysiological differences between the eyes open and eyes closed resting state conditions. It uses a straight forward and easily replicable approach to investigate the electrophysiology of baseline conditions in terms of energy efficiency. Furthermore, we introduce the method of persistent homology from algebraic topology to study network connectivity dynamics free of the threshold selection problem. The minimization of the wiring cost for functional connectivity networks acting over networks of intracranial electrodes provides a new avenue for understanding the electrophysiology of resting state.
