# Author Correction: Exploring the alpha desynchronization hypothesis in resting state networks with intracranial electroencephalography and wiring cost estimates

**DOI:** 10.1038/s41598-018-23049-3

**Published:** 2018-03-14

**Authors:** Jaime Gómez-Ramírez, Shelagh Freedman, Diego Mateos, José Luis Pérez Velázquez, Taufik A. Valiante

**Affiliations:** 1 The Hospital for Sick Children, Neurosciences and Mental Health program, Toronto, Canada; 2 Concordia University, Montreal, Canada; 3 Toronto Western Hospital, Krembil Research Institute, Toronto, Canada

Correction to: *Scientific Reports* 10.1038/s41598-017-15659-0, published online 15 November 2017

The original version of this Article contained a typographical error in the spelling of the author Taufik A. Valiante, which was incorrectly given as Taufik Valiante. This has now been corrected in the PDF and HTML versions of the Article.

